# Biomarkers for Early and Late Stage Chronic Allograft Nephropathy by Proteogenomic Profiling of Peripheral Blood

**DOI:** 10.1371/journal.pone.0006212

**Published:** 2009-07-10

**Authors:** Sunil M. Kurian, Raymond Heilman, Tony S. Mondala, Aleksey Nakorchevsky, Johannes A. Hewel, Daniel Campbell, Elizabeth H. Robison, Lin Wang, Wen Lin, Lillian Gaber, Kim Solez, Hamid Shidban, Robert Mendez, Randolph L. Schaffer, Jonathan S. Fisher, Stuart M. Flechner, Steve R. Head, Steve Horvath, John R. Yates, Christopher L. Marsh, Daniel R. Salomon

**Affiliations:** 1 Department of Molecular and Experimental Medicine, The Scripps Research Institute, La Jolla, California, United States of America; 2 Mayo Clinic, Scottsdale, Arizona, United States of America; 3 DNA Microarray Core, The Scripps Research Institute, La Jolla, California, United States of America; 4 Department of Chemical Physiology, The Scripps Research Institute, La Jolla, California, United States of America; 5 Department of Biostatistics, University of California, Los Angeles, California, United States of America; 6 The Texas Medical Center, Houston, Texas, United States of America; 7 University of Alberta, Edmonton, Canada; 8 St. Vincent Medical Center, Los Angeles, California, United States of America; 9 Scripps Center for Organ and Cell Transplantation, Scripps Health, La Jolla, California, United States of America; 10 Glickman Urological Institute, The Cleveland Clinic, Cleveland, Ohio, United States of America; Instituto Mario Negri, Italy

## Abstract

**Background:**

Despite significant improvements in life expectancy of kidney transplant patients due to advances in surgery and immunosuppression, Chronic Allograft Nephropathy (CAN) remains a daunting problem. A complex network of cellular mechanisms in both graft and peripheral immune compartments complicates the non-invasive diagnosis of CAN, which still requires biopsy histology. This is compounded by non-immunological factors contributing to graft injury. There is a pressing need to identify and validate minimally invasive biomarkers for CAN to serve as early predictors of graft loss and as metrics for managing long-term immunosuppression.

**Methods:**

We used DNA microarrays, tandem mass spectroscopy proteomics and bioinformatics to identify genomic and proteomic markers of mild and moderate/severe CAN in peripheral blood of two distinct cohorts (n = 77 total) of kidney transplant patients with biopsy-documented histology.

**Findings:**

Gene expression profiles reveal over 2400 genes for mild CAN, and over 700 for moderate/severe CAN. A consensus analysis reveals 393 (mild) and 63 (moderate/severe) final candidates as CAN markers with predictive accuracy of 80% (mild) and 92% (moderate/severe). Proteomic profiles show over 500 candidates each, for both stages of CAN including 302 proteins unique to mild and 509 unique to moderate/severe CAN.

**Conclusions:**

This study identifies several unique signatures of transcript and protein biomarkers with high predictive accuracies for mild and moderate/severe CAN, the most common cause of late allograft failure. These biomarkers are the necessary first step to a proteogenomic classification of CAN based on peripheral blood profiling and will be the targets of a prospective clinical validation study.

## Introduction

Kidney transplantation offers a significant improvement in life expectancy and quality of life for patients with end stage renal disease[1]. Unfortunately, a chronic, progressive allograft dysfunction of uncertain etiology continues to be a primary cause of graft loss[2], [3]. There has been some evolution of terminology to better describe the histological basis of this chronic, progressive nephropathy, commonly referred to as chronic allograft nephropathy (CAN) and more recently as interstitial fibrosis and tubular atrophy (IF/TA)[4]–[6]. Immunologic factors linked to IF/TA are acute, sub-clinical and chronic rejection, HLA mismatching and circulating donor-specific anti-HLA antibodies[7], [8]. Non-immunologic factors include hypertension, chronic toxicity of calcineurin inhibitors, hyperfiltration and diabetes mellitus[9]–[12]. The unifying mechanism is thought to be a progressive cycle of vascular and tissue injury, incomplete repair, compensatory hypertrophy, progressive interstitial fibrosis and nephron loss[13]. In the present paper, we will use the term CAN to refer to biopsy-documented and graded IF/TA in the absence of any other known cause[14].

As early as two years post kidney transplant, protocol biopsies have shown that more than 50% of recipients have mild CAN[2], [15], [16] and by 10 years over 50% of kidney transplant recipients have severe CAN that is associated with diminishing graft function[2]. Traditional kidney function measurements like serum creatinine and glomerular filtration rates used to predict CAN have poor predictive values[17] and a diagnosis requires a transplant biopsy[18], [19]. While critical to examine structural changes prior to graft loss, predicting graft outcomes strictly based on the kidney biopsy is difficult and this invasive procedure has significant costs and risks for patients. Thus, there is a pressing medical need to identify minimally invasive biomarkers that are able to identify early stages of CAN at a time that changes in therapy may alter outcomes.

Rapidly evolving technologies for genomics have created new opportunities to develop minimally invasive biomarkers. Recent studies, including our own, have identified genes that are differentially expressed at the mRNA level in kidney biopsies in the presence of CAN[16], [20], [21]. The limitation of these studies is that they require an invasive transplant biopsy. Others have successfully sampled urine and peripheral blood using RT-qPCR or proteomics to identify small numbers of potential biomarkers for CAN, though none are validated for clinical use[22], [23]. Here we report a set of unique gene and protein expression-based signatures for CAN measured in the peripheral blood that directly addresses the critical medical need for a set of minimally invasive biomarkers for this devastating and common complication of kidney transplantation. These signatures have a predictive accuracy of 80% for mild CAN and 92% for moderate/severe CAN. This is the first study using whole genome DNA microarrays and tandem mass spectrometry proteomics to successfully apply proteogenomics of peripheral blood to clinical transplantation.

## Materials and Methods

### Patient Populations

Test Set 1 comprised 42 kidney transplant patients randomized to either cyclosporine or de novo rapamycin at the Cleveland Clinic, whose clinical courses have been previously, described [15], [16], [24]. Density gradient-purified peripheral blood lymphocytes (PBL) were collected at the time of protocol two-year biopsies. Test Set 2 comprised 35 patients from 3 clinical centers (St. Vincent's Medical Center, Scripps Clinic, and Cleveland Clinic). All patients were on FK506. Whole blood was collected directly into PaxGene Tubes (PreAnalytix) at the time of biopsies for suspected CAN or protocol one-year biopsies. All the studies in this manuscript were covered by Human Subjects Research Protocols approved by each Center's Institutional Review Board and by the IRB of The Scripps Research Institute as the parent institution. Informed consent was obtained from all study subjects in the study.

### Pathology

Banff IF/TA grades based on tubulointerstitial features were determined for all patients by kidney biopsies: grade 0 (no evidence CAN), 1 (mild CAN), and 2 (moderate CAN) and 3 (severe CAN). We merged patients with Banff 2 and Banff 3 IF/TA to increase numbers. Diagnosis was done first by local pathologists and reviewed in a blinded fashion by Drs. Kim Solez (Set 1) and Lillian Gaber (Set 2). C4d staining was only available in the more recently acquired Test Set 2.

### Gene expression profiling and analysis

RNA was extracted from Test Set 1 using Trizol (Invitrogen) and in Test Set 2 using Paxgene Blood RNA system (PreAnalytix) and globin transcripts were reduced using GlobinClear (Ambion). Biotinylated cRNA was prepared using Ambion MessageAmp Biotin II (Ambion) and hybridized to Affymetrix Human Genome U133 Plus 2.0 GeneChips. Normalized signals that were generated using a quantile normalization strategy (RMAExpress[25]) were used for class comparisons (ANOVA) and class predictions (BRB Array Tools; http://linus.nci.nih.gov/BRB-ArrayTools.html). We chose the Diagonal Linear Discriminant Analysis (DLDA) method for class predictions, which is based on maximum likelihood discriminant rules that give consistently good results with our data set and others[26]. Receiver Operating Characteristics (ROC) analysis was done using JROCFIT (http://www.rad.jhmi.edu/jeng/javarad/roc/JROCFITi.html). Heatmaps were generated using Cluster and Treeview[27] and functional analysis was performed using Gene Ontology (GO) (http://www.geneontology.org/) and Ingenuity Pathway Analysis (IPA). Consensus analysis was designed to identify true classifiers in the two independently collected data sets. Variability between the two test sets within each class (i.e. Banff 1/Test Set 1 vs. Banff 1/Test Set 2) was eliminated by removing all genes with a Student's t-test p-value of <0.05 after which the remaining genes were used to identify consensus candidates by class comparisons. All differentially expressed gene lists are shown in Supplementary Data Tables S1, S2, S3, S4, S5, S6. All the microarray data for this study is available for review at the private GEO accession site http://www.ncbi.nlm.nih.gov/geo/query/acc.cgi?token=vbgvzkwuggqiqpy&acc=GSE12187.

### Shotgun LC/MS/MS proteomics

All protein samples were prepared from density gradient-purified PBL. Individual patient samples were pooled within each Test Set (3 samples/pool) based on Banff classifications and pools were run in triplicates. Total protein was proteolytically digested with trypsin and samples run using Multidimensional Protein Identification Tool (MudPIT) protocol as previously described[28] using an LTQ XL mass spectrometer (ThermoFisher). Raw data were searched against the EBI-IPI_human_12_01_2006 database supplemented with a decoy database where each entry of the original protein contains its reversed sequence. Database searching used SEQUEST (v27)[29] and outcomes were filtered using DTASelect[30]. Relative quantifications were done using spectral counts normalized to the median of the total spectral counts[31]. Pair-wise comparisons between CAN biopsy classes were done by differentially expressed proteins (Student's t-test, p≤0.05) and as all-or-none/unique events.

## Results

### Study Population

Recipients in both Test Sets were sex and age matched (Table 1). The only significant differences in Test Set 1 were Donor age between Banff 0 and Banff 1 groups. In Test Set 2 there were significant differences in induction therapy between Banff 0 and Banff 1 and between Banff 0 and the Banff 2,3; time to biopsy between Banff 0 and Banff 1 and between Banff 0 and the Banff 2,3; and steroid use between Banff 0 and Banff 1 and between Banff 0 and Banff 2,3. Only the Banff 2,3 group in Test Set 2 had a significantly higher serum creatinine compared to the Banff 0, thus, renal function levels per se were not a major determinant of the gene profiles. The higher creatinine levels in the Banff 2,3 group of Test Set 2 most likely reflect the fact that this group was “biopsy for cause”, while Test Set 1 were all protocol biopsies done regardless of any renal function change. However, by design, the two Test Sets differed significantly in recipient age, HLA mismatch, induction therapy, clinical center, immunosuppression, serum creatinines, and time to biopsy.

**Table 1:** Clinical Characteristics of the Study Populations.

	Test Set 1				Test Set 2				Test Set 1 vs. Test Set 2
	Banff 0	Banff 1	Banff 2,3	Significance	Banff 0	Banff 1	Banff 2,3	Significance	Significance
**Number**	18	15	9	NA	8	14	13	NA	NS
**Recipient age**	42.61±12.8	48.47±11.6	45.67±17.5	NS	56.88±12.2	51.36±12.6	49.08±12.9	NS	Banff0 = 0.01
**Recipient gender (% female)**	38.9	20	44.4	NS	62.5	35.7	53.8	NS	NS
**Recipient race African American**	22.22	13.33	11.11	NS	0	14.3	7.7	NS	NS
**Pre tx diabetes**	16.7	26.7	22.2	NS	25	14.3	7.7	NS	NS
**PRA>20% (%)**	5.6	6.7	11.1	NS	12.5	7.1	15.4	NS	NS
**HLA mismatch**	3.06±1.7	2.66±1.6	2.67±2.2	NS	3.43±2.4	4.33±1.4	3.58±1.6	NS	Banff1 = 0.008
**Deceased donor**	55.6	73.3	77.8	NS	75	71.4	46.2	NS	NS
**% re-transplant**	0	0	0	_	0	14.3	15.4	NS	NS
**Donor age**	32.39±15.7	42.33±11.8	37.11±12.1	Banff0 vs. Banff1 p = 0.05	31.25±19.3	41.54±17.7	44.62±13.4	NS	NS
**Donor gender female**	50	53.3	33.3	NS	12.5	57	53.8	NS	NS
**Donor race African American**	16.7	13.3	11.1	NS	0	7.1	7.7	NS	NS
**Induction**	100	100	100	NS	75	21.4	23.1	Banff0 vs. Banff1 p = 0.026; Banff0 vs. Banff2,3 p = 0.032	Banff1 = 0.0001 Banff2,3 = 0.0005
**Serum Creatinine**	1.32±0.38	1.45±0.51	1.84±0.77	NS	1.70±1.3	2.41±0.7	3.09±1.2	Banff0 vs. Banff2,3 p = 0.025	Banff1 = 0.0002 Banff2,3 = 0.007
**Time to Biopsy**	755±101	710±109	659±133	NS	420±309	1664±1364	2398±1120	Banff0 vs. Banff1 p = 0.005; Banff0 vs. Banff2,3 p = 0.00002	Banff0 = 0.05 Banff1 = 0.02 Banff2,3 = 0.0001
**CNI**	38.9	60	77.8	NS	100	100	84.6	NS	Banff0 = 0.007
**MMF**	100	93.3	88.9	NS	75	78.6	76.9	NS	NS
**Steroids**	100	100	100	NS	37.5	100	92.3	Banff0 vs. Banff1 p = 0.0002; Banff0 vs. Banff2,3 p = 0.0022	Banff1 = 0.0009
C4d± staining*	ND	ND	ND	NA	NA	2	3	NS	NA

ND - Not Done.

NA - Not Applicable.

NS - Not Significant.

### Gene expression profiling of mild CAN

**Figure 1:**
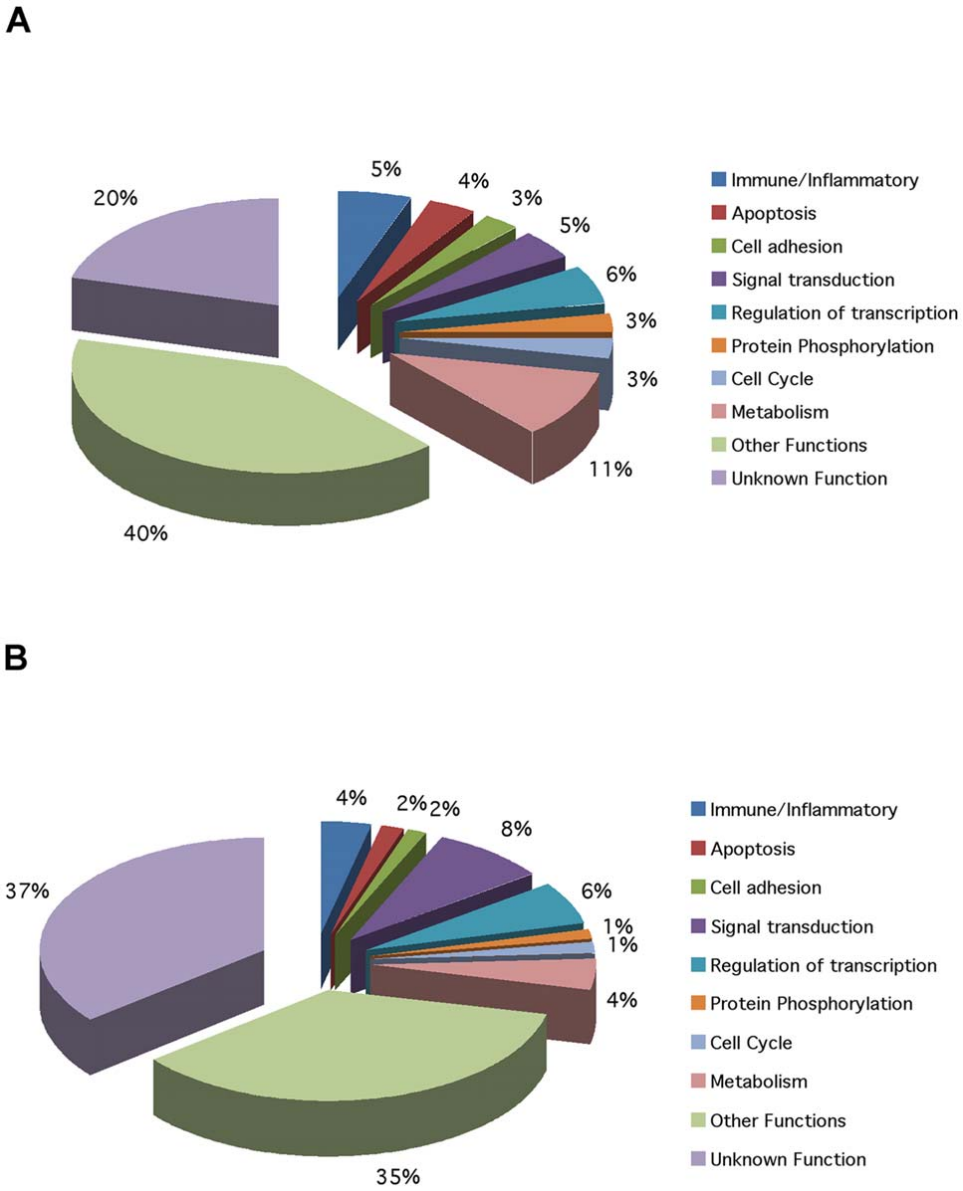
Pie charts showing the Gene Ontology annotations for both Test Sets for Banff 0 vs. Banff 1 (mild CAN). Each slice of the pie chart represents the percentage of genes represented by that functional class. A) Test Set 1 (PBL) for Banff 0 vs. Banff 1; B) Test Set 2 (Whole Blood) for Banff 0 vs. Banff 1. The first key point is that there is no difference in the general groups of differentially expressed, functional genes assigned in an unbiased fashion by analysis using Gene Ontology whether we are interrogating the profiles of PBL or whole blood. The second key point is that there are a number of genes representing different pathways connected to immune/inflammatory and tissue injury mechanisms.

We performed ANOVA-based class comparisons between Banff 0 (no histological evidence of CAN) and Banff 1 (mild CAN). At p-values <0.005, 1066 genes (1307 probe sets) were differentially expressed. Annotation of function by Gene Ontology (GO) shows 8 categories comprised of >25 genes each (Figure 1A) including 58 genes linked to immunity and inflammation. IPA shows that these 1066 genes fall into 27 networks with >15 genes per network (Supplementary Data Table S7). The top network was immune response and two additional networks in the top 10 were also immune response with 27 and 22 focus genes, respectively. The top canonical pathway was Toll-like Receptor Signaling followed by SAPK/JNK, Apoptosis, Notch and Death Receptor and Interferon Signaling. Finding 1066 significantly differentially expressed genes is a first indication that PBL transcript profiling is capable of classifying subjects defined by CAN biopsy histology. Class prediction using DLDA demonstrates 90% mean correct classification[32], [33]. Supervised hierarchical clustering shows misclassification of only 2 samples (Supplementary Figure S1).

Based on gene expression profiles of the whole blood samples in Test Set 2, there were 1429 genes (1591 probe sets) differentially expressed at p-values <0.005. GO annotation of gene functions revealed the same groups as PBL including 50 immune response genes (Figure 1B). IPA reveals 30 networks with ≥15 genes per network (Supplementary Data Table S8). The top canonical pathways were: B Cell Receptor, Toll-like Receptor, Death Receptor, Chemokine, Glucocorticoid Receptor, and IL-4 Signaling. DLDA demonstrates 88% mean correct classification. Supervised hierarchical clustering shows misclassification of only 1 sample (Supplementary Figure S2).

**Figure 2:**
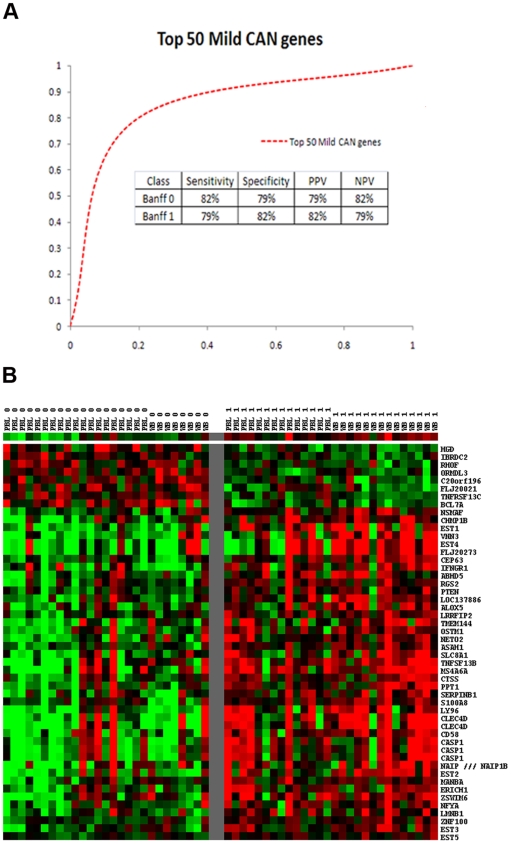
Class prediction analysis of Banff 0 vs. Banff 1 (mild CAN) based on Diagonal Linear Discriminant Analysis for the top 50 Banff 0 vs. Banff 1 consensus genes ranked by p values. A) depicts the Receiver Operating Characteristic (ROC) curves and provides the Sensitivity, Specificity, Positive Predictive Value (PPV) and Negative Predictive Value (NPV); B) depicts the heat map classifying Banff 0 vs. Banff 1 using the top 50 consensus genes where (red) is up-regulated and (green) is down-regulated.

A consensus analysis for Banff 0 vs. Banff 1 was performed with these two independently collected data sets by a class comparison at p-values <0.005 and identified 393 genes (424 probe sets) significantly differentially expressed in both data sets. This “consensus” gene list represents the intersection of these two significantly different test sets classifying mild CAN by blood transcription profiling. We then combined all the samples of both Test Sets (n = 55) and performed class predictions using the top 50 differentially expressed, consensus genes ranked by p values to obtain a class prediction accuracy of 80% depicted as a ROC curve (Figure 2A). Figure 2B shows the heat map classifying Banff 0 vs. Banff 1 using the 50 genes. The importance of the heat map display is that it provides the reader a clear look at the consistency of gene expression changes in all the samples studied for both test sets. It is clear that there are large “blocks” of up- or down-regulated genes that classify the Banff 0 vs. Banff 1 (mild CAN). However, it is also evident why signatures of multiple genes are necessary to achieve high class predictive accuracies in heterogenous clinical populations that are the reality of transplantation medicine. A logical question is how many genes are actually necessary for a robust diagnostic? We took the top 10 and top 3 genes from our consensus set for mild CAN and performed class prediction using the DLDA method. The top 10 had a predictive accuracy of 80%, sensitivity of 85% and specificity of 77%, whereas the top 3 genes had a predictive accuracy of 80%, sensitivity of 74% and specificity of 86%.

### Gene expression profiling of moderate/severe CAN

**Figure 3:**
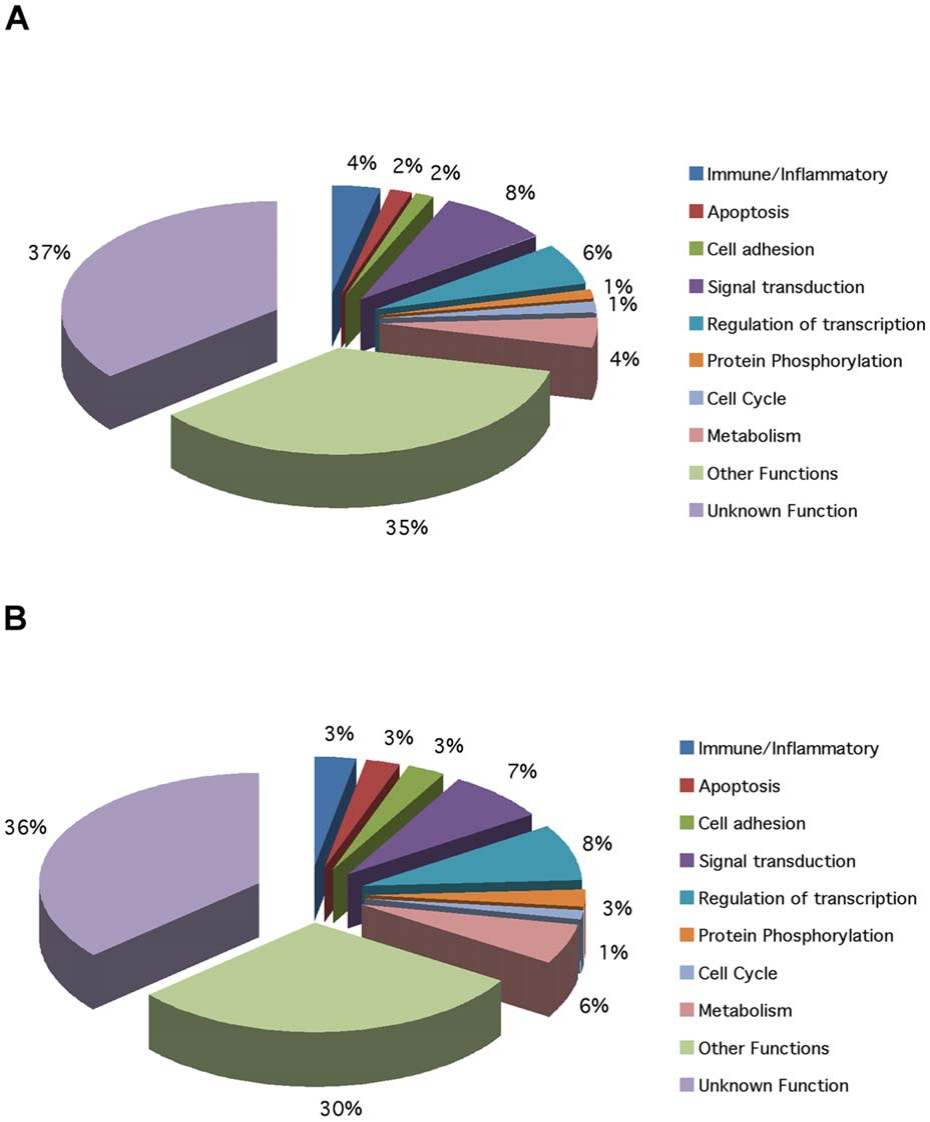
Pie charts showing the Gene Ontology annotations for both Test Sets for Banff 0 vs. Banff 2,3 (moderate to severe CAN). Each slice of the pie chart represents the percentage of genes represented by that functional class. A) Test Set 1 (PBL) for Banff 0 vs. Banff 2,3; B) Test Set 2 (Whole Blood) for Banff 0 vs. Banff 2,3. The first key point is that there is no difference in the general groups of differentially expressed, functional genes assigned in an unbiased fashion by analysis using Gene Ontology whether we are interrogating the profiles of PBL or whole blood (as was true for the Banff 0 vs. Banff 1 comparisons shown in Figure 1). The second key point is that the number of differentially expressed immune/inflammatory genes is significantly less than observed in mild CAN with many more genes linked to metabolic and other pathways consistent with the hypothesis that early stages of CAN are driven by immune/inflammatory mechanisms and tissue injury but later stages reflect slowly progressive renal dysfunction and fibrosis.

**Figure 4:**
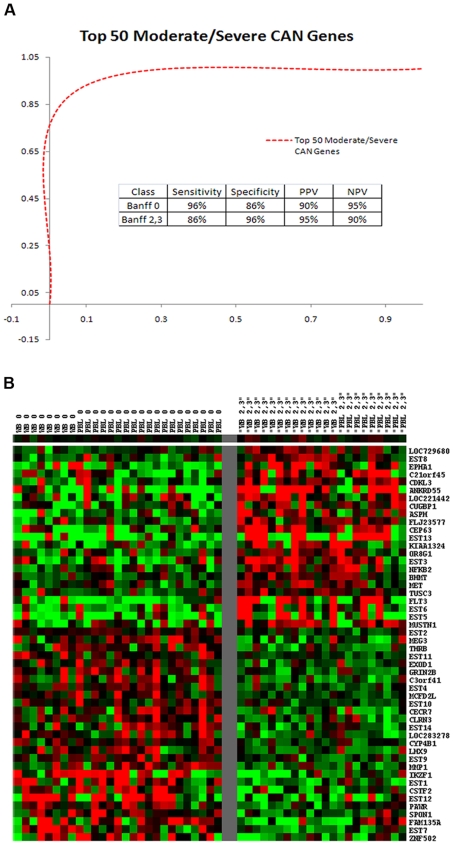
Class prediction analysis of Banff 0 vs. Banff 2,3 (moderate to severe CAN) based on Diagonal Linear Discriminant Analysis for the top 50 Banff 0 vs. Banff 2,3 consensus genes ranked by p values. A) depicts the Receiver Operating Characteristic (ROC) curves and provides the Sensitivity, Specificity, Positive Predictive Value (PPV) and Negative Predictive Value (NPV); B) depicts the heat map classifying Banff 0 vs. Banff 2,3 using the top 50 consensus genes where (red) is up-regulated and (green) is down-regulated.

Class comparisons between Banff 0 and Banff 2,3 identified genes differentially expressed between patients without CAN and those with moderate to severe CAN. In Test Set 1, 172 genes were differentially expressed (p<0.005) and classified the samples by DLDA with 78% accuracy. In Test Set 2 there were 545 differentially expressed genes. DLDA classified 95% of the samples accurately. Functional annotation by Gene Ontology (GO) is shown in Figure 3. A consensus analysis was done as already described to yield 62 differentially expressed genes (p<0.005) shared for both Test Sets of moderate/severe CAN (n = 49). The ROC curve for the top 50 genes from this consensus gene set shows a class prediction accuracy of 92% (Figure 4A). Figure 4B shows the Banff 0 vs. Banff 2,3 heat map using these 50 consensus genes. An attempt to make the same predictions with only the top 10 or top 3 ranked genes was not possible using DLDA for moderate/severe CAN.

### Proteomic expression of mild and moderate/severe CAN

To investigate using proteomics to define blood cell biomarkers for CAN, we performed shotgun tandem mass spectrometry. All samples represented purified PBL obtained at the same time as biopsies. We did not use the whole blood samples from Test Set 2 because high quality protein preparations cannot be obtained from PaxGene tubes. Differential protein expression was performed using a relative quantification strategy based on normalized spectral counts[31].

We identified 206 differentially expressed proteins (p<0.05) for Banff 0 vs. Banff 1 (mild CAN). In addition, we identified 135 proteins unique to Banff 0 and 167 proteins unique to Banff 1. Class comparisons for Banff 0 vs. Banff 2,3 (moderate/severe CAN) yielded 282 differentially expressed proteins (p<0.05) and 509 proteins unique to Banff 2,3. We found 95 proteins differentially expressed in mild and moderate/severe CAN as compared to Banff 0, representing candidate protein markers for any stage of CAN. In parallel, 94 proteins were differentially expressed only in mild CAN and these were linked to cell death, cell signaling, and post-translational protein modifications. The 168 proteins differentially expressed only in moderate/severe CAN were linked to cellular morphology, growth and proliferation and signaling via ERK/MAPK, acute phase responses, IGF1 and PPARa/RXRa.

There were 135 proteins unique to mild CAN and 322 proteins unique to moderate/severe CAN. Both mild and moderate/severe CAN had immune and inflammation related proteins (20 and 37, respectively) but many of these proteins are not mapped to the same functional pathways (e.g. calcium signaling in mild CAN and apoptosis, NK cell and PTEN signaling for moderate/severe CAN). In other cases, such as signaling via T and B cell receptors, IL4 and JAK/STAT, the same canonical pathways were found but different unique proteins were identified.

Using only the differentially expressed proteins, DLDA obtained a 64% mean correct classification of mild CAN and an 83% correct classification for moderate/severe CAN. In contrast, the unique proteins identified only in the blood of patients with biopsy-documented mild (n = 135) or moderate/severe CAN (n = 322), represent candidate biomarkers with a 100% class prediction value in this data set.

We compiled the matches between proteins identified by mass spectrometry and mRNA transcripts identified using microarrays. The premise is that protein/transcript matches are a form of candidate biomarker validation based on two independent technologies. There were 11 matches for the 393 consensus genes for mild CAN, 32 matches for the 1066 genes for mild CAN in Test Set 1 and 40 matches for the 1429 genes for mild CAN in Test Set 2. There were no matches for the 62 consensus genes for moderate/severe CAN but 9 matches in the 172 genes for moderate/severe CAN in Test Set 1 and 9 matches in the 545 genes for moderate/severe CAN in Test Set 2. All protein/transcript matches are listed in Supplementary Data Table S9.

## Discussion

The primary objective of this study was the discovery of biomarkers in the peripheral blood of kidney transplant patients with biopsy-documented interstitial fibrosis and tubular atrophy (IF/TA) and no known cause, which we refer to here as Chronic Allograft Nephropathy (CAN)[14]. To this end, we purposely integrated the results of two, independently collected sets of patient samples that were significantly different in multiple clinical elements. Thus, the selection of biomarker candidates is not significantly influenced by the time of biopsy (ranging from 1 to 6 years post-transplant), the specific immunosuppressive protocols (use of different calcineurin inhibitors vs. sirolimus) or the technology used to purify the mRNA transcripts (density gradient-separated cells vs. whole blood). This experimental design was chosen for its advantages in defining a consensus set of robust candidate biomarkers for CAN suitable for clinical use.

We acknowledge that it is likely that using more closely matched sets of patient samples, for example, patients only 2 years post-transplant or only one source of blood cell RNA such as the PaxGene tubes, would result in higher total numbers of differentially expressed candidate mRNA transcripts and proteins. We remind the reader again that our use of PBL-derived RNA in Test Set 1 reflected the best approach possible at a time before whole blood RNA analysis using the PaxGene technology was possible. However, despite these limitations, our predictions of correct classifications for CAN based on the consensus mRNA candidates described here for these otherwise very heterogeneous clinical data sets are 80% for mild CAN and 92% for moderate/severe CAN. In this context, the widely used prostate specific antigen (PSA) biomarker, tested in an equally heterogenous human population, was originally introduced with a predictive value of 28–35%[34] based on the rationale that there was no other minimally invasive option for early detection of prostate cancer at that time, which is true for CAN today.

There are two critical questions for the design of the next study as a prospective serial blood monitoring trial. First, we are almost certain that restricting the analysis to whole blood samples obtained using the PaxGene system will significantly increase the number of consensus genes in any two test set comparison that is done. However, while some will demand that the next study use more standardized selection criteria for subjects (for example, identical immunosuppressive therapy), for detailed biopsy histology (for example, identical grades of IFTA based on the Banff schema) and a single time point post transplant for the biopsy evaluations, our view is that the reality of current clinical practice is remarkably diverse and that is not going to change. In a clinical situation, the best biomarker signature is the one least dependent for classification accuracy on any kind of homogenous selection criteria. What would be the general value of a set of biomarkers that were only useful at exactly two years post-transplant or only applicable to patients with a single grade of IFTA on the biopsy? In that context, we believe that the current experimental design, encompassing so much of the diversity currently present in clinical transplantation practice in the US, is actually the best design for biomarker discovery.

A question that cannot be answered yet is how many biomarkers are necessary to insure a robust diagnostic test. Our results here indicate that whole genome profiling is certainly not necessary as we obtain very reasonable predictive accuracy, sensitivity and specificity with 150, 100 and 50 total genes per signature. There are now several technology platforms perfectly suitable for point of clinical service implementation that can measure 100 genes or more cost effectively and within hours. It may also be possible to do such testing with even fewer genes but we suspect that the complexity of transplant populations and clinical phenotypes will frustrate efforts to reduce the testing signature to such a minimal set. As for application to clinical practice, we propose that the model will be serial, prospective measurements of the signature at regular intervals for the life of the kidney transplant. The absence of a positive CAN/IFTA signature at any point in time will indicate adequate immunosuppression or over-immunosuppression. Careful reductions in immunosuppressive drug doses could then be used with repeat monitoring of the signature to establish the optimal drug combination and level for each patient to prevent CAN/IFTA and insure the long term safety of the therapy.

Biomarker discovery has been done successfully using peripheral blood profiling for acute rejection in heart transplantation[35], [36]. Peripheral blood studies of kidney transplant patients with “operational tolerance” included 22 patients with biopsy-documented chronic rejection[37]. Two of the genes (DPYD, IRS2) reported to distinguish “operational tolerance” are identified in our consensus sets. Our earlier study of 42 kidney biopsies showed that gene expression profiles of CAN had significant up-regulation of immune/inflammation, fibrosis and tissue remodeling genes[16]. However, only 5 genes from these CAN biopsies were identified in the current peripheral blood consensus sets. A study of 11 CAN biopsies identified 3 genes linked to immunity and fibrosis that were tested by quantitative PCR in urine and peripheral blood with good correlations in urine but none in peripheral blood[38]. In our study of acute rejection, candidate mRNA transcripts were identified in both biopsies and peripheral blood, but with no overlap[24]. Therefore, candidate gene biomarkers identified in peripheral blood appear to be distinct from those identified in tissue. It is not our position that gene expression profiling of the biopsy is not useful but rather that the biopsy and the peripheral blood are very different compartments and simple comparisons of the two are not informative.

Urine based proteomics have been used to identify biomarkers for acute rejection using SELDI-TOF mass spectroscopy[23], [39] but to our knowledge this is the first study to identify blood cell-based proteomic markers for transplantation using tandem mass spectroscopy. We have identified several hundred proteins that are significantly differentially expressed in peripheral blood of patients with CAN as a function of histology grade, mild to moderate/severe. However, the group of uniquely identified proteins potentially represents the highest value biomarker candidates though this will require validation in another independent set of samples. Integrating proteomics with gene expression, we identified over 80 protein/transcript matches for CAN providing candidate validation based on two independent technologies. On the other hand, using the differentially expressed protein and transcript matches did not significantly improve the classifications obtained with the consensus gene expression set alone (data not shown). It is important to note that in such complex clinical samples that we can interrogate greater than 80% of the cell's transcriptome but only about 10% of the proteome. However, technologies to increase the capability of tandem mass spectrometry proteomics to identify and quantify candidates are rapidly evolving and other technologies such as antibody arrays and fluorescent bead assays are also potential platforms for clinical implementations. Moreover, as already noted, the potential of the uniquely expressed proteins identified for mild CAN (n = 135) and moderate/severe CAN (n = 322) are to be 100% diagnostic of the graft histology. Testing these predictions in the next study will be of critical importance.

We purposefully did not make any effort in this study to compare the results of the gene expression profiles of the transplant biopsies that were all done in parallel with the peripheral blood gene expression profiling. The objective of this work was to prove the hypothesis that proteogenomic profiling of peripheral blood could yield a set of minimally invasive biomarkers capable of diagnosing the presence and severity of CAN with high confidence and without the necessity of an invasive kidney biopsy. While we understand the mechanistic importance of understanding the gene and protein changes in the kidney that occur with progression of CAN, this is an entirely separate question and will be the subject of another manuscript. The central challenge addressed in the present study was the fidelity of proteogenomic profiling of the blood compartment. In fact, we propose that the peripheral blood represents a fully functional and distinct compartment of the immune system that actively serves to traffic and modulate all the components of effector immunity. While clearly the tissue injury that causes the progression of CAN is occurring in the kidney, we believe that a significant determinant of the phenotype of the host immune response, either acceptance of the graft or chronic rejection, is actually established and subsequently regulated within the peripheral blood compartment, lymph nodes and spleen.

Finally, it is important to emphasize that it was not evident to anyone in transplantation at the beginning of our study that there would be a molecular signature in peripheral blood cell mRNA or cellular proteins for the chronically progressive kidney transplant dysfunction referred to by the term CAN/IFTA. We recognized the complications in the genetic and clinical diversity of transplant patients, multiple clinical centers, the cellular complexity of peripheral blood, and the impact of factors such as immunosuppression, environment and time post-transplant. Nonetheless, we have discovered several hundred mRNA and proteomic biomarkers in peripheral blood defining unique proteogenomic signatures and demonstrated correlations with histologically mild (80% class prediction accuracy for top 50 gene candidates) and moderate/severe CAN (92% class prediction accuracy for top 50 gene candidates). Thus, this study represents a clear proof of concept for the use of peripheral blood biomarkers as diagnostic tools for clinical transplantation and specifically, for CAN.

We are not concluding that our current gene sets are the optimal final candidates for clinical implementation, due to the technical limitations discussed above created by using PBL vs. whole blood assays. However, we do conclude that our results are the basis for the next critical step, a prospective clinical trial in kidney transplantation with serial blood monitoring and genome-wide gene expression and proteomic profiling. Another key point is that the design of the present study was all based on profiling subjects with biopsy-proven CAN and that was the correct design for a biomarker discovery program and an initial validation. Thus, the evidence presented supports the conclusion that these candidate genes are diagnostic. On the other hand, the point of doing the serial monitoring study next with clinical and biopsy-confirmed transplant outcomes will be to test the hypothesis that peripheral blood gene expression profiling is also capable of predicting the development of CAN. The importance of this next step is that if these biomarker panels are proven to be as predictive as they are now diagnostic, then the logical next question will be whether we can use these gene expression signatures to manage and optimize the efficacy and safety of immunosuppression for patients on an individual basis. This will introduce personalized medicine to kidney transplantation.

## Supporting Information

Table S1 (0.23 MB XLS)

Table S2 (0.32 MB XLS)

Table S3 (0.06 MB XLS)

Table S4 (0.13 MB XLS)

Table S5 (0.10 MB XLS)

Table S6 (0.03 MB XLS)

Table S7 (0.04 MB XLS)

Table S8 (0.04 MB XLS)

Table S9 (0.92 MB XLS)

Table S10 (0.05 MB XLS)

Figure S1 (7.45 MB TIF)

Figure S2 (7.45 MB TIF)
