# Supplementary figures and images for: Biomarkers for Early and Late Stage Chronic Allograft Nephropathy by Proteogenomic Profiling of Peripheral Blood
Source: PLoS One. 2009 Jul 10;4(7):e6212. doi: 10.1371/journal.pone.0006212 (PMC2703807; doi:10.1371/journal.pone.0006212)

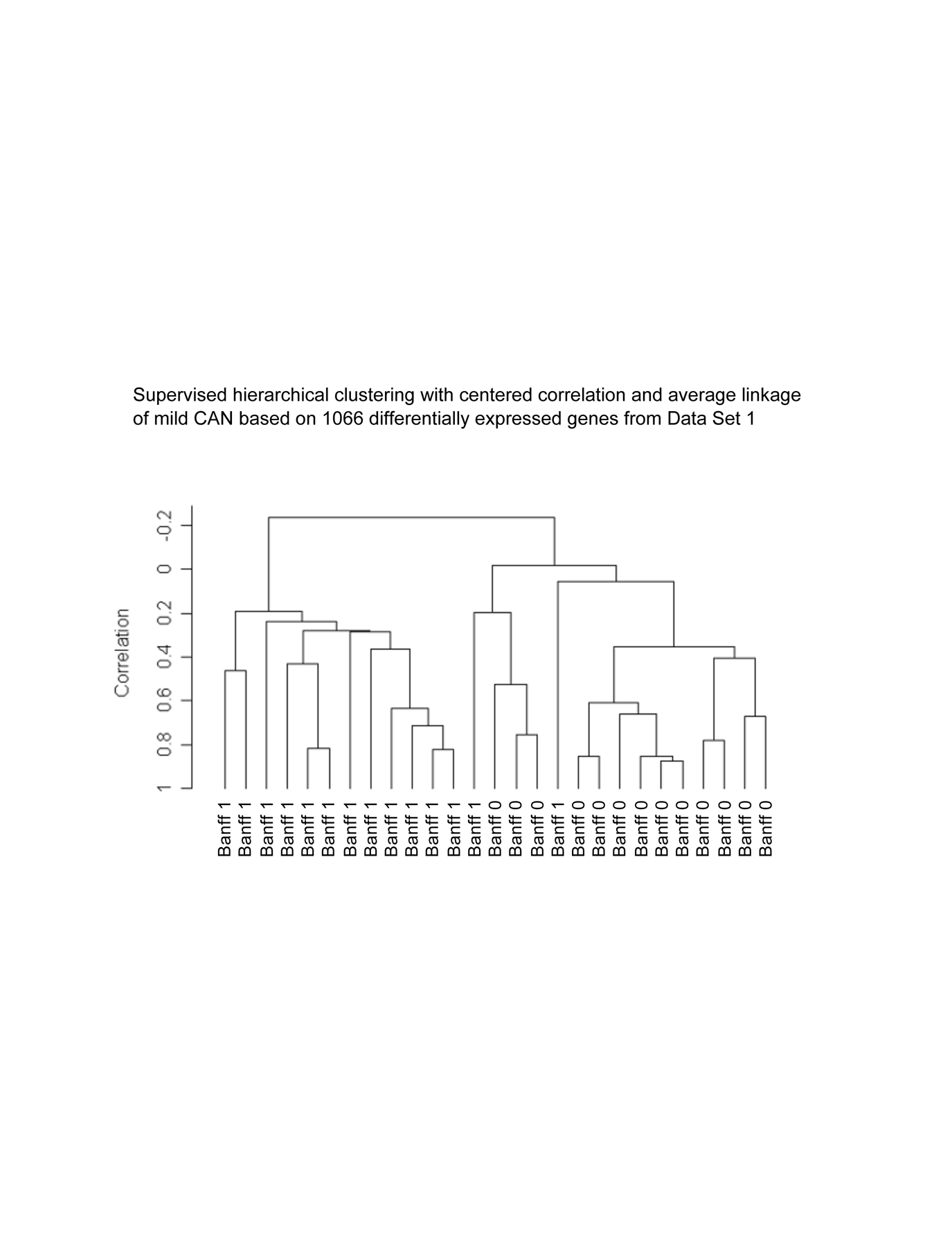

Supplement: Figure S1 — (7.45 MB TIF) [file pone.0006212.s011.tif]

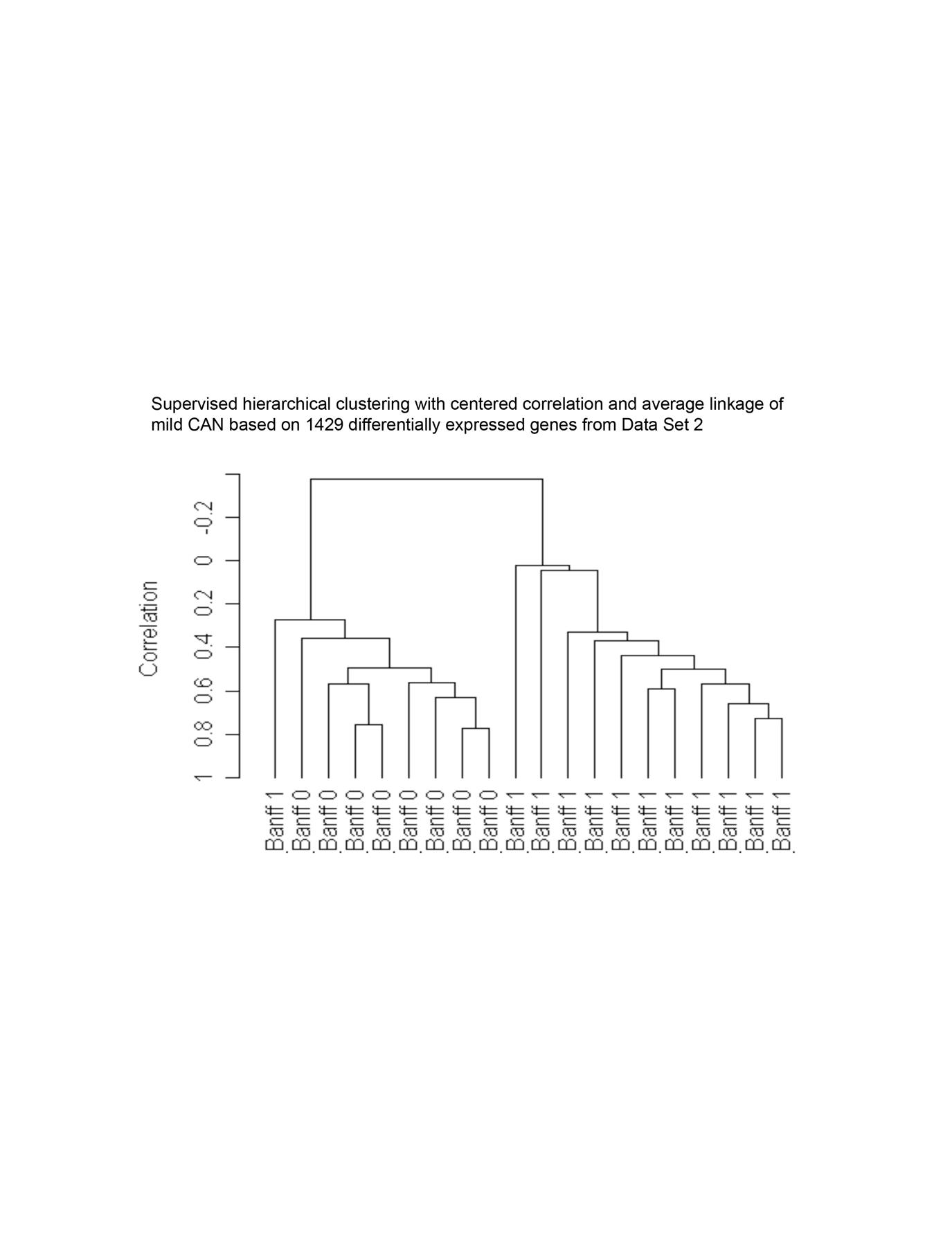

Supplement: Figure S2 — (7.45 MB TIF) [file pone.0006212.s012.tif]
